# Improved DC and RF Characteristics of GaN-Based Double-Channel HEMTs by Ultra-Thin AlN Back Barrier Layer

**DOI:** 10.3390/mi15101220

**Published:** 2024-09-30

**Authors:** Qian Yu, Chunzhou Shi, Ling Yang, Hao Lu, Meng Zhang, Xu Zou, Mei Wu, Bin Hou, Wenze Gao, Sheng Wu, Xiaohua Ma, Yue Hao

**Affiliations:** 1 State Key Discipline Laboratory of Wide Band-Gap Semiconductor Technology, School of Microelectronics, Xidian University, Xi’an 710071, China; 2 ZTE Corporation, Shenzhen 518057, China

**Keywords:** ultra-thin barrier layer, double channel, GaN HEMT, InAlN barrier, AlN barrier, radio frequency

## Abstract

In order to improve the off-state and breakdown characteristics of double-channel GaN HEMTs, an ultra-thin barrier layer was chosen as the second barrier layer. The strongly polarized and ultra-thin AlN sub-barrier and the InAlN sub-barrier are great candidates. In this article, the two epitaxial structures, AlGaN/GaN/AlN/GaN (sub-AlN) HEMTs and AlGaN/GaN/InAlN/GaN (sub-InAlN) HEMTs, were compared to select a more suitable sub-barrier layer. Through TEM images of the InAlN barrier layer, the segregation of In components can be seen, which decreases the mobility of the second channel. Thus, the sub-AlN HEMTs have a higher output current density and transconductance than those of the sub-InAlN HEMTs. Because the high-quality AlN barrier layer shields the gate leakage current, a 294 V breakdown voltage was achieved by the sub-AlN HEMTs, which is higher than the 121 V of the sub-InAlN HEMTs. The current gain cut-off frequency (*f*_T_) and maximum oscillation frequency (*f*_max_) of the sub-AlN HEMTs are higher than that of the sub-InAlN HEMTs from low to high bias voltage. The power-added efficiency (PAE) and output power density (*P*_out_) of the sub-AlN HEMTs are 57% and 11.3 W/mm at 3.6 GHz and 50 V of drain voltage (*V*_d_), respectively. For the sub-InAlN HEMTs, the PAE and *P*_out_ are 41.4% and 8.69 W/mm, because of the worse drain lag ratio. Thus, the *P*_out_ of the sub-AlN HEMTs is higher than that of the sub-InAlN HEMTs.

## 1. Introduction

GaN HEMTs have been successfully applied to 5G base stations due to their high breakdown field strength and high electron mobility [1,2,3]. In order to further improve the output current density and high linearity of GaN HEMTs, double-channel devices have been widely considered as a potential alternative technology for single-channel devices [4,5]. Double-channel devices can distribute carriers across multiple channels [6], providing additional carriers to increase output current density [7]; double-channel devices widen the gate voltage swing (GVS) to improve the linearity of GaN HEMTs [8]. However, due to the long distance of the second channel from the gate, the control of the channel by the gate is weakened, resulting in poor off-state characteristics and poor subthreshold swing [9]. In order to solve this issue, it is effective to reduce the barrier layer thickness of the second channel. However, once the conventional AlGaN barrier layer is thinned, its polarization strength will be affected, resulting in a low concentration of carriers in the second channel [10]. Therefore, the strongly polarized barrier layers InAlN and AlN are good alternatives [11,12].

The InAlN barrier layer has the advantage of strong polarization, and the 0.17 indium component InAlN has no lattice mismatch with GaN [13]. However, the segregation of In components introduces additional scattering of alloying impurities, which affects mobility and RF performance. The segregation of the indium component of the ultra-thin InAlN barrier layer is particularly strong [14,15]. Especially when the InAlN barrier layer is used as the barrier layer of the second channel, the alloy disorder scattering and interfacial rough scattering will not only affect the mobility of the second channel but also affect the mobility of the upper channel [16,17]. Thus, the advantages of the AlN barrier layer as an ultra-thin second channel barrier layer are reflected because the alloy disorder scattering and roughness scattering are reduced by the AlN barrier layer [18]. High mobility of the AlN barrier can be achieved. Although the lattice mismatch between the AlN barrier layer and the GaN channel layer is large, a high-quality AlN layer can be achieved when the thickness of the AlN barrier layer is under critical thickness [19]. The AlN layer is also a strongly polarized barrier layer; high carrier concentration can be realized [20]. Compared with the InAlN barrier layer, AlN is more suitable as the second channel barrier layer of the double-channel device.

To improve the gate control of the second channel, the most suitable second channel ultra-thin barrier layer was discussed by comparing two types of hybrid double-channel structures, AlGaN/GaN/InAlN/GaN (sub-InAlN) and the AlGaN/GaN/AlN/GaN (sub-AlN) HEMTs. Both structures can significantly improve the subthreshold swing characteristics of GaN HEMTs. However, the characterization of material quality can be seen using a Transmission Electron Microscope (TEM) and the electrical performance of devices was compared using DC testing, small signal testing, and large signal testing. These results can be reflected in the negative impact on the double-channel GaN HEMTs of the ultra-thin InAlN barrier layer and the segregation of In components.

## 2. Device Structure and Fabrication

The epitaxial layers of InAlN HEMTs (AlN HEMTs) were grown on a 3-inch SiC substrate by metal–organic chemical vapor deposition (MOCVD), consisting of a 1 μm GaN buffer layer, a 400 nm unintentionally doped GaN channel layer, a 3 nm InAlN (AlN) barrier layer, a 10 nm unintentionally doped GaN channel layer and a 20 nm AlGaN barrier layer from bottom to top.

**Figure 1:**
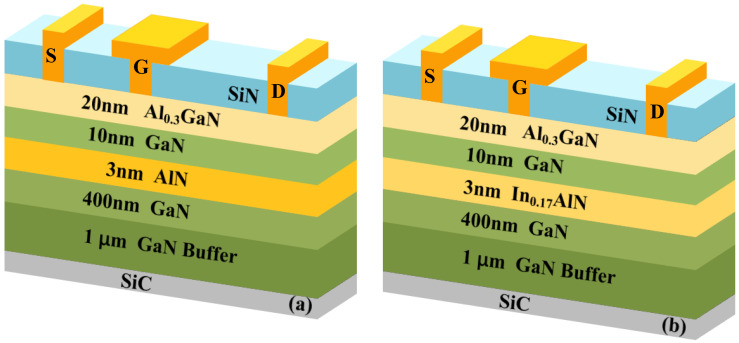
Schematic diagram of the epitaxial layer structure of (**a**) AlGaN/GaN/AlN/GaN and (**b**) AlGaN/GaN/InAlN/GaN.

The device fabrication process started with depositing a metal stack including Ti/Al/Ni/Au. To form the ohmic contact, the device was annealed at 860 °C for 60 s in a N_2_ atmosphere. Then, nitrogen ion implantation was used to form the device’s electrical isolation. Then, the device performed SiN passivation by Plasma-Enhanced Chemical Vapor Deposition (PECVD). Afterward, lithography and CF_4_-based plasma etching were used to define the 0.5 μm gate window and the 1.3 μm gate cap, and a T-shaped gate was achieved with a Ni/Au metal stack for Schottky contact. Finally, the interconnection of the device was achieved by the Ti/Au metal stack. The source–drain spacing (*L*_sd_) of the device is 5 μm. The gate width and gate length (*L*_g_) are 2 × 50 μm and 0.5 μm, respectively. The cross-sectional structures of the two devices are shown in Figure 1a,b.

**Figure 2:**
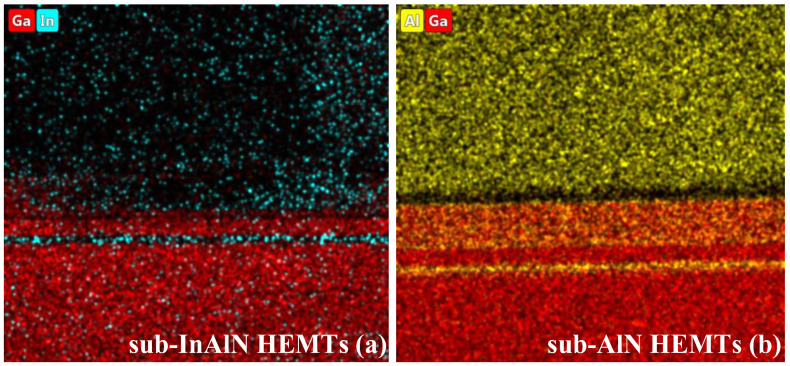
The energy-dispersive X-ray spectroscopy mapping images of In, Al, and Ga element distribution.

The indium/gallium (In/Ga) element distribution of the sub-InAlN HEMTs and the aluminum/gallium (Al/Ga) element distribution of the sub-AlN HEMTs are shown in the energy-dispersive X-ray spectroscopy mapping images in Figure 2a and 2b, respectively. As we can see from Figure 2a, the segregation of In components in the sub-InAlN HEMTs is very strong. The uniform Al component in the sub-AlN HEMTs is shown in Figure 2b.

## 3. Results and Discussion

**Figure 3:**
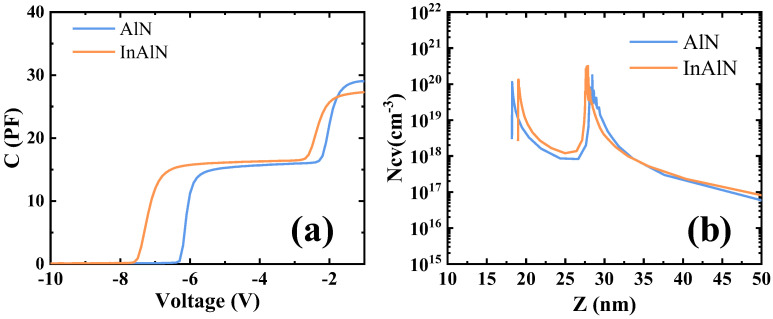
(**a**) CV and (**b**) electron concentration distribution of the AlGaN/GaN/InAlN/GaN and AlGaN/GaN/AlN/GaN HEMTs.

**Figure 4:**
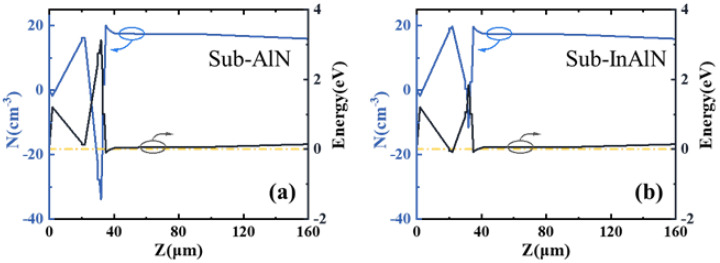
The energy band diagram and electron concentration distributions of the (**a**) sub-AlN HEMTs and (**b**) sub-InAlN HEMTs.

Figure 3a shows the capacitance–voltage characteristics of the sub-InAlN HEMTs and the sub-AlN HEMTs. Due to the double channel, there are two obvious platforms in the CV curves. The electron concentration distribution curve of the two kinds of HEMTs is shown in Figure 3b. Due to the segregation of In components, the coupling effect of the sub-InAlN HEMTs is stronger than that of the sub-AlN HEMTs. Thus, the centroid of 2DEG of the sub-InAlN located in the upper channel is affected to shift downward [21]. Since the upper channel structure is the same on both HEMTs, the electron concentration is the same. The coupling effect between the two channels of the sub-InAlN HEMTs is stronger than that of the sub-AlN HEMTs. The coupling effect is the change of electron transport mode in the upper channel [22]. This is the reason why the electron concentration in the second channel of the sub-InAlN HEMTs is higher than that of the sub-AlN HEMTs. The band diagram is simulated by the Silvaco TCAD shown in Figure 4. This is similar to the Ncv curve.

**Figure 5:**
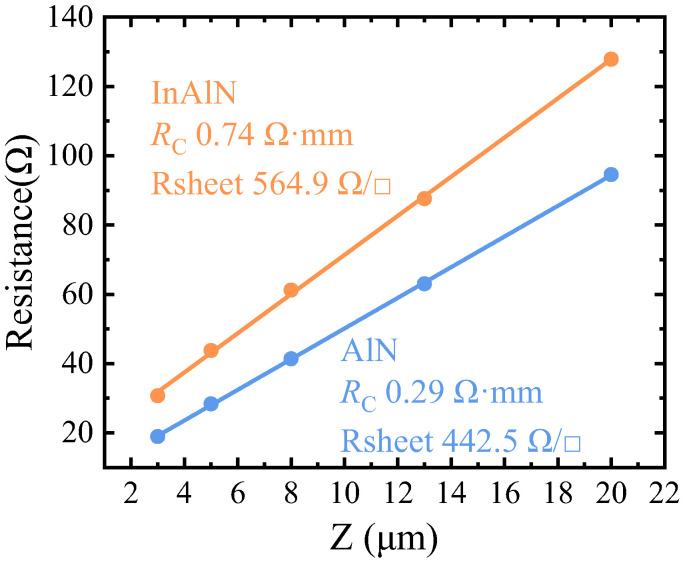
The contact resistance (*R*_c_) and block resistance (*R*_sheet_) of the sub-InAlN/sub-AlN HEMTs tested from the transmission line model (TLM).

The contact resistance (*R*_c_) and sheet resistance (*R*_sheet_) of the sub-InAlN and sub-AlN HEMTs are shown in Figure 5. The *R*_c_ and *R*_sheet_ of the sub-AlN HEMTs are 0.29 Ω·mm and 442.5 Ω/□, respectively. The *R*_c_ and *R*_sheet_ of the sub-InAlN HEMTs are 0.74 Ω·mm and 564.9 Ω/□, respectively. These results are obtained from the transmission line model (TLM) testing according to the following formula:

(1) $$R_{\mathrm{sheet}}=\frac{\rho_{c}}{L_{T}^{2}}$$

(2) $$R_{C}=\frac{R_{sheet}\cdot L_{T}}{W_{C}}$$

*ρ*_c_, *L_T_*, and *W*_C_ are the contact resistivity, transmission length, and length of metal electrodes, respectively. Due to the lower mobility of the sub-InAlN HEMTs, the *R*_sheet_ of the sub-InAlN HEMTs is higher than that of the sub-AlN HEMTs. According to Formula (2), the lower *R*_C_ was achieved by the sub-AlN HEMTs because of the better *R*_sheet_.

**Figure 6:**
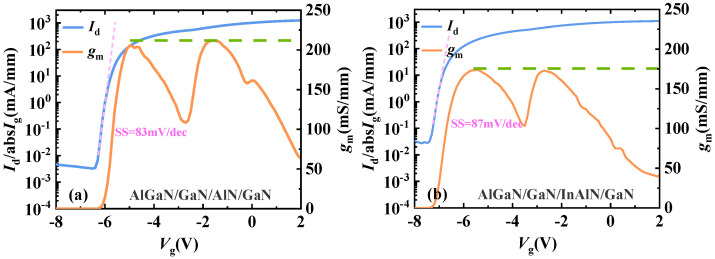
The transfer curve of (**a**) AlGaN/GaN/AlN/GaN and (**b**) AlGaN/GaN/InAlN/GaN HEMTs.

The transfer curve of the sub-InAlN HEMTs and the sub-AlN HEMTs is shown in Figure 6a,b. Due to the ultra-thin lower barrier layer, the gate control on the lower channel is enhanced. The subthreshold swing of the sub-AlN HEMTs and the sub-InAlN HEMTs is 83 mV/dec and 87 mV/dec, respectively. Compared to conventional double-channel GaN HEMTs, both the sub-AlN HEMTs and the sub-InAlN HEMTs have a lower subthreshold swing. This is due to the ultra-thin second barrier layer, which improves gate control of the second channel. The first *g*_m_ peak of the sub-AlN HEMTs is 220 mS/mm, which is higher than 173 mS/mm for the sub-InAlN HEMTs. This is because the mobility of the second channel in the sub-AlN HEMTs is higher than that of the sub-InAlN HEMTs. The off-state drain leakage current of the sub-AlN HEMTs is an order of magnitude lower than that of the sub-InAlN HEMTs. The reason why off-state current leakage of the sub-AlN HEMTs is lower than that of the sub-InAlN HEMTs is the segregation of In components [23].

**Figure 7:**
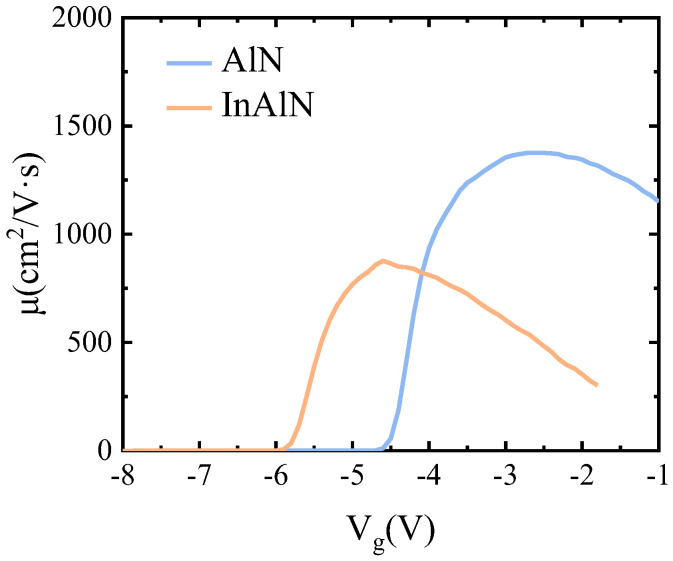
The mobility curve of AlGaN/GaN/InAlN/GaN and AlGaN/GaN/AlN/GaN HEMTs extracted from the transfer curve.

The mobility of the second channel of the sub-InAlN HEMTs and the sub-AlN HEMTs is shown in Figure 7. The second channel’s mobility is extracted from the transfer curve, which was tested by the fatFET device at a *V*_d_ of 0.1 V. The mobility was measured by the following equation [24]:

(3) $$\mu_{FE}=\frac{g_{m}V_{DS}}{C_{d}\frac{W}{L}{\left(V_{DS}-R_{t}I_{D}\right)}^{2}}$$

where *C*_d_ is the gate–drain capacitance per unit area, *R*_t_ is the group of all gate voltage independent resistors, and *L* and *W* are the gate length and width. The peak measured mobility of the sub-InAlN and sub-AlN HEMTs was 876 and 1375 cm^2^/V·s, respectively. Due to the higher *g*_m_ of the second channel in the sub-AlN HEMTs, the second channel’s mobility in the sub-AlN HEMTs is higher than that of the sub-InAlN HEMTs. Due to the segregation of In components, alloy disorder scattering and interfacial rough scattering affect the mobility of the upper channel [16,17]. The higher mobility of the sub-AlN HEMTs results in high RF performance.

**Figure 8:**
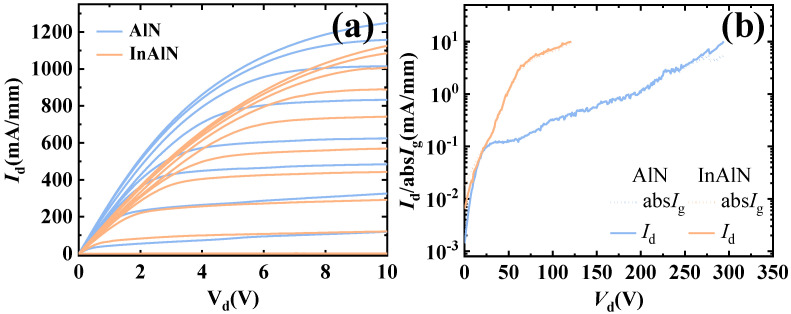
(**a**) The output curve of the sub-AlN/sub-InAlN HEMTs. (**b**) The breakdown characteristics of the sub-AlN/sub-InAlN HEMTs.

The output current curve of the sub-AlN and sub-InAlN HEMTs is shown in Figure 8a. The output current density of the sub-AlN HEMTs is 1248 mA/mm, which is higher than 1150 mA/mm of sub-InAlN HEMTs. The higher saturation current (*I*_d,max_) of the sub-AlN HEMTs results in a higher output power density. The knee voltage of the sub-AlN HEMTs is 5 V, which is lower than the 7 V of the sub-InAlN HEMTs. Due to the lower *R*_sheet_ of the sub-InAlN HEMTs, a better knee voltage was realized by the sub-AlN HEMTs. The breakdown characteristics of the sub-AlN and sub-InAlN HEMTs are shown in Figure 8b. The breakdown voltage (*V*_br_) of the sub-AlN HEMTs and the sub-InAlN HEMTs is 294 V and 121 V. The *V*_br_ is defined as 10 mA/mm. Due to In precipitation, the leakage current of the channel is increased, resulting in the degradation of the breakdown voltage of the sub-InAlN HEMTs [23]. The RF output power density can be increased by the high breakdown voltage of the sub-AlN HEMTs.

**Figure 9:**
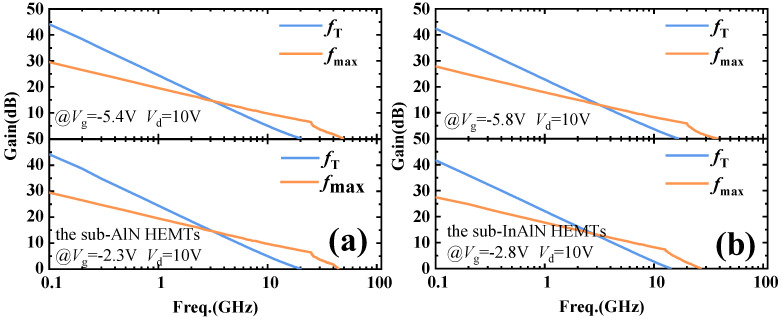
The *f*_max_ and *f*_T_ of (**a**) the sub-InAlN and (**b**) sub-AlN HEMTs at a *V*_d_ of 10 V.

The small signal characteristics of the sub-InAlN/sub-AlN HEMTs were measured by the Agilent E8361 network analyzer. Corresponding to the transconductance peak *V*_g_, the maximum oscillation frequency (*f*_max_) and the current gain cut-off frequency (*f*_T_) are measured at a *V*_d_ of 10 V, which is shown in Figure 9a,b. For the sub-AlN HEMTs, the *f*_T_ and *f*_max_ are 20.5 GHz and 50 GHz at a *V*_g_ of −5.4 V, corresponding to the first *g*_m_ peak; the *f*_T_ and *f*_max_ are 20.3 GHz and 45 GHz at a *V*_g_ of −2.3 V, corresponding to the second *g*_m_ peak. For the sub-InAlN HEMTs, the *f*_T_ and *f*_max_ are 17 GHz and 38.4 GHz at a *V*_g_ of −5.8 V, corresponding to the first *g*_m_ peak; the *f*_T_ and *f*_max_ are 14.4 GHz and 27.3 GHz at a *V*_g_ of −2.8 V, corresponding to the second *g*_m_ peak. It can be seen that the *f*_T_ and *f*_max_ of the sub-AlN HEMTs are higher than that of the sub-InAlN HEMTs. This is because both *g*_m_ peaks of the sub-AlN HEMTs are higher than the two *g*_m_ peaks of the sub-InAlN HEMTs.

**Figure 10:**
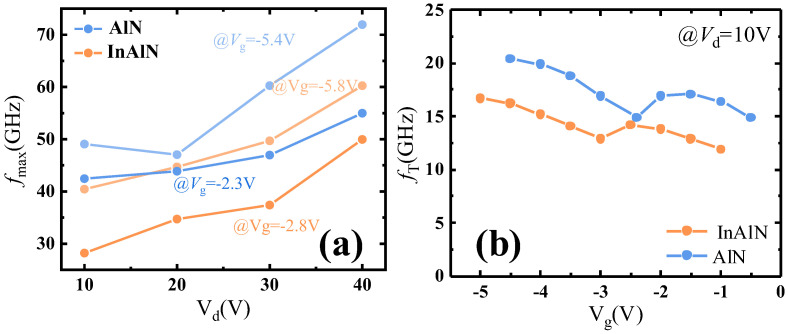
(**a**) The *f*_max_ of the sub-InAlN/sub-AlN HEMTs versus *V*_d_. (**b**) The *f*_T_ of the sub-InAlN/sub-AlN HEMTs versus *V*_g_.

As shown in Figure 10a, the *f*_max_ of the sub-InAlN/sub-AlN HEMTs was measured at the gate voltage of the two transconductance peaks from 10 V to 40 V of *V*_d_, respectively. Corresponding to the different gate voltage of the *g*_m_ peak, the *f*_max_ of the sub-AlN HEMTs is higher than that of the sub-InAlN HEMTs. The current gain cut-off frequency *f*_T_ of the sub-InAlN HEMTs and the sub-AlN HEMTs versus *V*_g_ is shown in Figure 10b, which was measured at 10 V of *V*_d_. The formula for *f*_T_ and *f*_max_ is as follows:

(4) $$f_{T}=\frac{g_{m}^{*}}{2\pi C_{g}}$$

(5) $$f_{\max}=\frac{f_{T}}{2\sqrt{\frac{R_{g}+R_{i}+R_{s}}{R_{ds}}+2\pi f_{T}R_{g}C_{gd}}}$$

where $g_{m}^{*}$ is the intrinsic *g_m_*, *C*_g_ is the capacitance of the gate, *C*_gd_ is the capacitance of the gate–drain, and *R*_g_, *R*_i_, *R*_s_, and *R*_ds_ are the resistance of the gate, intrinsic *g_m_*, source, and source–drain. The trend of the *f*_T_ curve as a function of gate voltage is the same as that of the transfer curve. Under a series of gate biases, the *f*_T_ of the sub-AlN HEMTs is higher than that of the sub-InAlN HEMTs.

**Figure 11:**
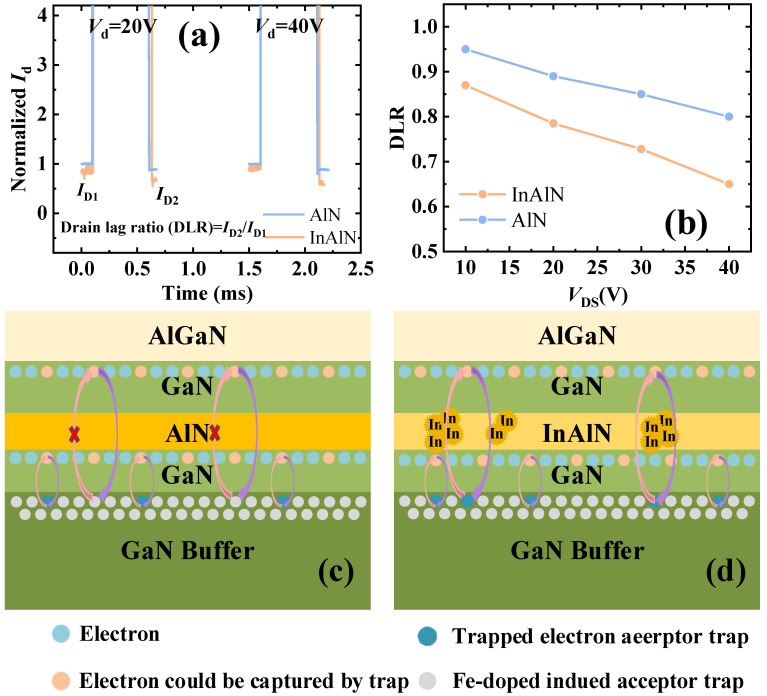
(**a**) The drain lag ratio (DRL) of the sub-AlN/sub-InAlN HEMTs. (**b**) The DRL versus *V*_DS_. Schematic of AlN barrier suppressing the DRL in (**c**) sub-AlN HEMTs compared with the (**d**) sub-InAlN HEMTs.

To characterize the ability of the GaN buffer layer trap to capture channel electrons, the drain lag ratio (DLR) of the sub-InAlN and the sub-AlN HEMTs was measured at the drain quiescent biases of 0 V. In Figure 11a, the DLR test results for the sub-InAlN/AlN HEMTs are tested with a test pulse width of 500 μs. At 10 V and 20 V of *V*_d_, the DLR of the sub-InAlN HEMTs is 72% and 55%, and that of the sub-AlN HEMTs is 89% and 80%, respectively. Due to the segregation of In components, the sub-InAlN HEMTs have a worse drain lag delay than the sub-AlN HEMTs. In addition, the DLR of the sub-InAlN HEMTs deteriorates faster as *V*_d_ increases, which is shown in Figure 11b. Thus, the sub-AlN HEMTs are more suitable for high voltage than the InAlN HEMTs. The lower DLR of the sub-AlN HEMTs results in higher power-added efficiency.

As shown in Figure 11c,d, for the sub-InAlN HEMTs, the electrons in the first channel are more easily tunneled into the GaN buffer layer and captured by traps, because of the segregation of In components. The high-quality AlN layer can avoid the carriers in the first channel being captured by the trap in the GaN buffer. This also confirms that the sub-AlN HEMTs are more suitable for high-voltage applications than the sub-InAlN HEMTs [25].

**Figure 12:**
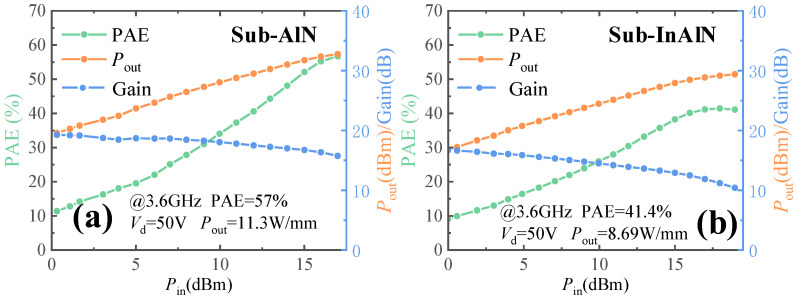
The load-pull measurement for the (**a**) sub-AlN and (**b**) sub-InAlN HEMTs at 3.6 GHz with 50 V of *V*_d_.

To prove the RF performance of the GaN HEMTs, the load-pull measurement was tested at 3.6 GHz and continuous wave (CW). The test results of the 50 V drain voltage are shown in Figure 12. The power-added efficiency and the output power density of the sub-InAlN HEMTs are 41% and 8.7 W/mm, respectively. The power-added efficiency and the output power density of the sub-AlN HEMTs are 57% and 11.3 W/mm, respectively. The power-added efficiency and the output power density of the sub-AlN HEMTs are greater than those of the sub-InAlN HEMTs. Due to the higher *I*_d,max_, the output power density of the sub-AlN HEMTs is higher than that of the sub-InAlN HEMTs. The reason why the sub-AlN HEMTs have higher PAE is that the sub-AlN HEMTs have greater DLR than the sub-InAlN HEMTs. The higher PAE of the sub-AlN is achieved due to the lower gate leakage current and knee voltage. Therefore, ultra-thin AlN is more suitable for the second channel barrier layer of double-channel GaN HEMTs than ultra-thin InAlN.

## 4. Conclusions

Due to the ultra-thin AlN/InAlN lower barrier layer, the off-state and subthreshold swing are improved. To determine which barrier layer is more suitable, the electrical properties of two strongly polarized barrier layers, InAlN and AlN, are compared. The TEM images of sub-InAlN HEMTs show that the ultra-thin InAlN layers are uneven and that the ultra-thin AlN layers have high quality. Therefore, the sub-AlN HEMTs have higher transconductance, output current density, and breakdown voltage. In RF performance, sub-AlN HEMTs have higher *f*_T_ and *f*_max_. The large signal characteristics of the sub-AlN HEMTs are also better than those of the sub-InAlN HEMTs. In summary, AlN is more suitable as the lower barrier layer for double-channel HEMTs than InAlN.

## Data Availability

The original contributions presented in the study are included in the article, further inquiries can be directed to the corresponding authors.
